# Editorial: 15 years of Frontiers in Human Neuroscience: social cognition and discourse processing

**DOI:** 10.3389/fnhum.2024.1506988

**Published:** 2024-10-28

**Authors:** Patricia A. Prelock, Vittorio Tantucci

**Affiliations:** ^1^Communication Sciences and Disorders, University of Vermont, Burlington, VT, United States; ^2^Lancaster Arts, Lancaster University, Lancaster, United Kingdom

**Keywords:** social cognition, neurogenic disorders, discourse processing, mental terms, metaphor processing

Social cognition and discourse processing are fascinating Research Topics in human neuroscience as they help explain the ways in which an individual makes sense of the social information in their environment. Over the past 15 years, Frontiers in Human Neuroscience (FHN) featured pivotal research on social cognition and discourse processing (Nolte et al., 2013), integrating usage-based and experimental methods for the analysis of social, developmental, metaphorical and multilingual communication (e.g., Cervenka et al., 2011; Lakoff, 2014; Verga and Kotz, 2013). The Journal contributed to expanding the scope of how we perceive and understand social cognition (Richlan, 2012; Tantucci, 2021; Tantucci and Wang, 2021) and the role of both experimental and socially embedded stimuli in typical and neurodiverse communication (Bonneh et al., 2011; Clough and Duff, 2020, but see also Prelock and Nelson, 2012; Tantucci and Wang, 2023).

The four articles featured in this Research Topic area contribute to broadening experimental, naturalistic and applied research into speech processing as they explore what we know about written discourse performance in people with acquired neurogenic communication disorders, how metaphoric meaning is accessed using event-related potentials in Chinese college students, the importance of language assessment to understand social cognition in children with ADHD and autism, and the use of narratives in adults with traumatic brain injury.

*Written discourse in diagnosis for acquired neurogenic communication disorders: current evidence and future directions* by Kim et al. reviews research over the last 20+ years to determine discourse differences that might differentiate patients with Alzheimer's disease, aphasia, mild cognitive impairment and primary progressive aphasia from one another and from non-patient populations. Although there is substantial evidence of numerous linguistic features in acquired neurogenic communication disorders that impact their social cognition and affect their ability to express themselves in both oral and written form, the evidence is not definitive in differentiating discourse abilities among the different clinical populations.

Psycholinguistic models of metaphor processing are examined in *An ERP study on the late stage of Chinese metaphor processing* by Xu et al.. Using conventional metaphors, familiarized metaphors and literal expressions, event-related potentials (ERPs) were elicited with probes comprised of semantically related words, literal meaning words and unrelated or non-words. Results revealed that unrelated and literal meaning words led to more negative waveforms than semantically related words and there was no difference between conventional and familiarized metaphors indicating metaphorical meaning can be directly accessed.

Language is central to the development of social cognition, and social deficits are defining criteria for neurodevelopmental disorders such as autism spectrum disorder and attention-deficit/hyperactivity disorder (ADHD). Further, social cognition and discourse processing are constructs that depend on capacity in several developmental domains such as cognition, language, emotional competence, behavior, and motor. In *Transdiagnostic considerations are critical to understanding childhood neurodevelopmental disorders* by Hoza and Shoulberg, the authors argue that there is dissatisfaction with current categorical diagnostic systems pushing the field to consider more transdiagnostic assessment approaches. Since mental health, language and cognitive delays appear early in childhood, children with neurodevelopmental disorders should be screened for language challenges first so that assessment approaches consider the child's basic linguistic capacity in the diagnostic process.

Finally, *Wishes, beliefs, and jealousy: use of mental state terms in Cinderella retells after traumatic brain injury* by Greenslade et al. explores the impact of traumatic brain injury (TBI) on social communication and specifically mental state terms (MST) in storytelling. Since social cognitive difficulties negatively impact relationships, addressing these difficulties is critical to patients achieving meaning in their daily activities. The authors investigated mental state term use in narrative retells of adults with and without TBI. Results indicated that fewer MSTs occurred in complex story retells but this appeared related to a lack of story content. The authors propose important implications for assessing and treating individuals with TBI.

Navigating social interactions, forming impressions, attributing intentions and emotions to others, understanding metaphors and retelling stories are important components to social cognition and discourse processing. Researchers have employed diverse methodologies to unravel the underlying cognitive and neural mechanisms of social cognition to shed light on how individuals make sense of the social world.

As these articles demonstrate, examining the intersection of social cognition and discourse processing deepens our understanding of how context shapes our social interaction and social communication. This research has implications for improving our understanding of the mechanisms that challenge children with neurodevelopmental disabilities and adults with acquired neurogenic disorders. It also reminds us of the importance of early assessment and intervention that differentiate the unique needs of the individual child or adult.
